# Efficacy and Tolerability of Fitostimoline (Vaginal Cream, Ovules, and Vaginal Washing) and of Benzydamine Hydrochloride (Tantum Rosa Vaginal Cream and Vaginal Washing) in the Topical Treatment of Symptoms of Bacterial Vaginosis

**DOI:** 10.5402/2012/183403

**Published:** 2012-10-30

**Authors:** F. Boselli, E. Petrella, A. Campedelli, M. Muzi, V. Rullo, L. Ascione, R. Papa, G. Saponati

**Affiliations:** ^1^Department of Obstetrics and Gynecology, University Hospital, 4121 Modena, Italy; ^2^Unità Operativa Complessa del Dipartimento Materno Infantile, Azienda Sanitaria Locale Napoli 1, Distretto Sanitario No. 50, Miano, 80145 Napoli, Italy; ^3^ISPharm s.r.l., Via dei Pubblici Macelli 194, 55100 Lucca, Italy

## Abstract

Two hundred and 91 patients showing signs and symptoms of bacterial vaginosis (BV) were randomized to receive topical treatment with Fitostimoline (vaginal cream and vaginal ovules + vaginal washing) or benzydamine hydrochloride (vaginal cream + vaginal washing) for 7 days. Signs (leucorrhoea, erythema, oedema, and erosion) and symptoms (burning, pain, itching, vaginal dryness, dyspareunia, and dysuria) (scored 0–3) were evaluated at baseline and at the end of treatment; the total symptoms score (TSS) was also calculated. In 125 patients, a bacterial vaginosis was confirmed by vaginal swab test. The primary efficacy variable analysis, that is, the percentage of patients with therapeutic success (almost complete disappearance of signs and symptoms), demonstrated that Fitostimoline ovules and vaginal cream were therapeutically equivalent and that pooled Fitostimoline treatment was not inferior to benzydamine hydrochloride. All the treatments were well tolerated, with only minor local adverse events infrequently reported. The results of this study confirmed that gynaecological Fitostimoline is a safe and effective topical treatment for BV.

## 1. Introduction

Bacterial vaginosis (BV; nonspecific vaginitis) is the most common outpatient vaginal disease in women of childbearing age [1]. Its pathogenesis and sexual transmission (if any) are still unclear [2]. BV is judged as an inflammatory process associated with the disruption of the normal vaginal lactate producing bacteria (mainly species of *Lactobacillus* genus) followed by an overgrowth of *Gardnella* and other anaerobic species [3]. Strong sexual activity, use of tight clothes, vaginal douching, and prolonged exposure to local irritants (i.e., scented soap) are considered as risk factors for BV [4, 5]. Symptoms of BV include a change in the amount, colour, or smell of normal vaginal discharge and vulvovaginal discomfort (irritation, itching, and burning). An increased local pH (>4.5) and a positive whiff-test of the vaginal fluid support the diagnosis of BV. A vaginal swab test is usually recommended to exclude a specific vaginitis caused by *Candida* or *Trichomonas* species. Importantly, BV can be associated with serious sequelae such as abortion, preterm delivery, and increased susceptibility to HIV infection and other sexually transmitted infections [4].

The watery extract of *Triticum vulgare*, the active ingredient of Fitostimoline (Farmaceutici Damor S.p.A., Naples-Italy), increases the healing processes both in cutaneous and noncutaneous tissues [6, 7]; gynaecological formulations of Fitostimoline (vaginal cream, ovule, and vaginal washing) are currently approved and marketed for the topical treatment of different inflammatory and dystrophic processes of the female reproductive system.

This study was performed to evaluate the efficacy and safety of Fitostimoline 20% vaginal cream, 600 mg ovules, and 40% vaginal washing in comparison with benzydamine hydrochloride 0.5% vaginal cream and 0.1% vaginal washing (Tantum Rosa, Angelini S.p.A., Italy). Benzydamine hydrochloride is a local anti-inflammatory and analgesic agent [8] marketed worldwide for the symptomatic treatment of gynaecological conditions and widely used in the topical treatment of bacterial vaginosis [9].

## 2. Patients and Methods

### 2.1. Patients

Women aged between 16 and 70 years with evidence of BV were enrolled; patients entered the study if they had at least two subjective symptoms and two objective signs (at least of moderate degree) of vaginal inflammation. Other gynaecological diseases (in addition to BV), immunosuppressive diseases (i.e., HIV infection), or treatments with antibiotics, anti-inflammatory agents, analgesics, antineoplastic, or immunosuppressive drugs in the previous 10 days were considered as exclusion criteria. Patients were asked to avoid vaginal sexual intercourse during the entire study period.

### 2.2. Study Design and Treatments

The study was performed according to an open-label, controlled, randomized, multicentre, 3-arm (Fitostimoline cream, Fitostimoline ovules, and Tantum Rosa cream) parallel design. The selection of an open-label design was due to the use of different pharmaceutical forms during the study. At the baseline visit, eligible patients were randomly assigned to receive one of the following treatments for 7 consecutive days: Fitostimoline vaginal cream + Fitostimoline vaginal washing, Fitostimoline ovules + Fitostimoline vaginal washing or Tantum Rosa vaginal cream + Tantum Rosa vaginal washing. Vaginal creams and ovules were applied in the evening, vaginal washings in the morning. Concomitant treatments with systemic or topical antibiotics, anti-inflammatory agents, or analgesics were not allowed. A vaginal swab test was performed at the baseline visit; in case of specific pathogen organisms growth, patients were treated after the 7-day control visit with antibiotics or antimycotics according to the result of the antibiogram. For ethical reasons, experimental treatments were started before the results of vaginal swab were available (some days required). Signs and symptoms of vaginitis were evaluated during the baseline visit and then after 7 days of treatment. A third follow-up visit 7 days after the end of treatment was performed only in those patients with negative vaginal swab and an incomplete symptoms improvement (≥60%). 

### 2.3. Outcome Measures

Six subjective symptoms (burning, pain, itching, vaginal dryness, dyspareunia, and dysuria) and 4 objective signs (leucorrhoea, vulvar erythema, vulvar oedema, and presence of abrasion/erosion) of vaginitis were evaluated by a semiquantitative scale (0 = absence; 1 = mild; 2 = moderate; 3 = severe). Vaginal pH measurement and whiff-test were performed at the baseline. The primary efficacy endpoint was the percentage of patients with *therapeutic success*, defined as resolution of signs and symptoms of vaginitis (total symptoms score < 2) at the end of treatment. For the overall assessment of clinical outcome (resolution, improvement, and failure: see the following), the results at the end of treatment were taken into account, except for those patients requiring a further follow-up visit.

The secondary efficacy variables were (1) the evolution of signs and symptoms of vaginitis, defined as the percentage of patients with resolution (overall score ≤ 2), improvement (decrease of the overall score versus baseline ≥ 50%), or failure (decrease of the overall score < 50%) and (2) the change from the baseline of the semiquantitative score, expressed for both single signs and symptoms and their sum (total symptoms score, TSS). Adverse events occurrence and vital signs (blood pressure, heart rate, body temperature) were evaluated as safety parameters.

### 2.4. Ethics

Informed consent was signed by all participants before any study-related procedure. The study was approved by the reference ethic committees of the study sites.

### 2.5. Statistics

The sample size was calculated on the hypothesis of a therapeutic success rate of 95% in all groups, with a hierarchical order of comparison Fitostimoline cream versus Fitostimoline ovules and then pooled Fitostimoline versus Tantum Rosa. The two Fitostimoline forms were considered therapeutically equivalent if the 95% CI of their difference was between −10% and +10%. Once that the therapeutic equivalence between Fitostimoline cream and ovules was demonstrated, the main efficacy comparison was performed between the pooled Fitostimoline group and Tantum Rosa. A limit of −10% was defined for the noninferiority of Fitostimoline versus Tantum Rosa. A sample size of approximately 100 patients per group was calculated to have a power of 90% of rejecting a hypothesis of nonequivalence or inferiority, respectively, at a level of significance of 0.025 in a one-sided test. The following populations were considered for data analysis: *intention to treat* (ITT), that is, all randomized patients; *restricted intention to treat* (ITTr), which included all randomized patients with at least one postbaseline control; *per protocol* (PP), that is, all ITT population who do not have any major protocol violation; *safety population* which included all randomized patients who received at least one application of the study medication. The evaluation of primary efficacy endpoint was performed in both the ITTr and in the PP population, while the analysis of the secondary efficacy endpoints was performed in the ITTr population only; safety variables were analyzed in the safety population. The comparison between groups of categorical variables was performed by using the Chi-square or the Fisher exact test, while comparisons between groups of the TSS were performed by using an ANCOVA model on final values, with treatment as factor and the baseline value as covariate. For continuous or continuous-like variables, changes between groups were analyzed by calculating the mean change from baseline and the corresponding 95% confidence interval (CI).

## 3. Results

The baseline demographic and clinical characteristics of the 291 women enrolled in the study (ITT population) are reported in Table 1. No significant differences between groups were found.

**Figure 1:**
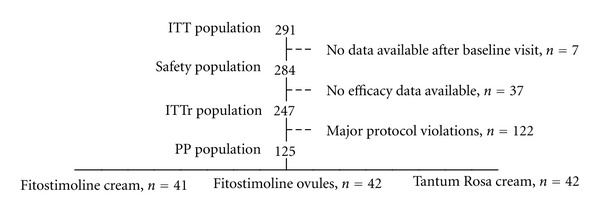
Study populations.

Seven patients were lost at the followup and were excluded from the safety population. A total of 37 patients were excluded from the ITTr population being efficacy data unavailable. One hundred and 25 patients completed the study without any major protocol violation (PP), while 122 were prematurely discontinued, mainly due to the growth of specific pathogen organisms in the vaginal swab (Figure 1).

Most patients (91.1% ITT) completed the study at the 7-day final visit, while a third follow-up visit was required by 3.4% of patients only. In 10 patients (3.4%), the final visit was anticipated, while 7 patients (2.4%) did not perform any further evaluation after the baseline visit.

The therapeutic success (PP population) was obtained in 41/41 (100%) and 41/42 (97.6%) of patients treated with Fitostimoline cream and ovules, respectively, with a difference between groups of 2.38% (2.33% to 6.99% 95% CI). Therefore, the two Fitostimoline groups were considered therapeutically equivalent, and all Fitostimoline-treated patients were pooled and compared with Tantum Rosa group.

### 3.1. Primary Efficacy Variable

Comparative results for the pooled Fitostimoline and the Tantum Rosa group for the primary efficacy endpoint (percentage of patients with therapeutic success) are reported in Table 2 for both the ITTr and the PP populations. The percentage of therapeutic success was 98.8% in the Fitostimoline group and 92.8 in the Tantum Rosa group (PP population), 82.8% and 78.6%, respectively, in the ITTr population. The lower confidence limit of the difference was above the predefined value of −10% in both the PP (−2.20) and the ITTr population (−6.26): therefore the treatment with Fitostimoline (vaginal cream and ovules) was not inferior to Tantum Rosa vaginal cream.

In the patients with positive vaginal swab, the percentage of therapeutic success was substantially similar with Fitostimoline (66.3%) and Tantum Rosa (67.5%), with a nonsignificant difference (−1.25%) between groups (95% CI −19.08 to 16.58).

### 3.2. Secondary Efficacy Variables

The outcome of vaginitis in term of resolution, improvement, or failure was slightly better, but not statistically different, in the Fitostimoline group (Table 3.)

**Figure 2:**
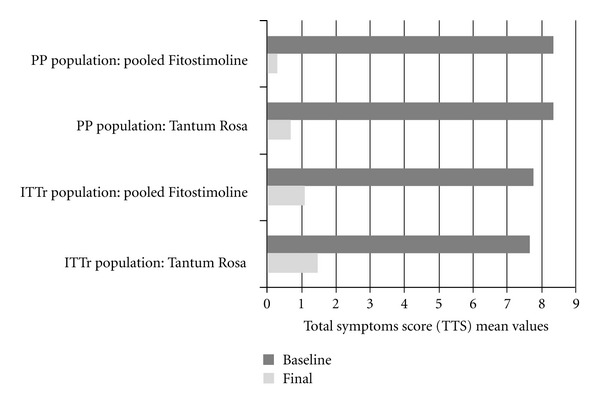
Total symptoms score (TTS) at the baseline and at the final visit.

The TSS values for both PP and ITTr population are reported in Figure 2. A significant decrease of the TSS versus baseline was observed in both groups; the comparison between Fitostimoline and Tantum Rosa did not reveal a significant difference in the ANCOVA test (*P* = 0.35).

Similar results were obtained for the scores of each individual signs and symptoms that decreased from the baseline with both treatments without significant differences between groups.

No serious adverse events were reported; nonserious mild adverse events (vaginal burning, vaginal discharge, and vaginal bleeding) were observed at application site in 4 patients (1.4%) in the whole, 2 (1.1%) treated with Fitostimoline, and 2 (2.0%) treated with Tantum Rosa, without significant difference between groups (*P* = 0.610). 

## 4. Conclusions

As the microbiological etiology of BV has not been completely defined [4], the role of nonantibiotic treatments should be continuously and carefully evaluated in randomized, controlled clinical trials. Three different “non-antibiotic” therapeutic approaches for BV were evaluated in the present study. The evening application of Fitostimoline ovules or vaginal cream, both associated with a morning Fitostimoline vaginal washing, induced a high percentage of clinical success (97.6% and 100%, resp.) and the two pharmaceutical forms were considered therapeutically equivalent. The pooled Fitostimoline and the benzydamine hydrochloride (Tantum Rosa vaginal cream + vaginal washing) treatments were effective in reducing the subjective symptoms and the objective signs of BV, without significant differences between group. The analysis of the primary efficacy endpoint (percentage of patients with therapeutic success) demonstrated the noninferiority of topical treatment of Fitostimoline in comparison with benzydamine hydrochloride. Even in the patients with “specific” vaginitis, a positive effect of both Fitostimoline and benzydamine hydrochloride was demonstrated, with a percentage of therapeutic success of 66.3% and 67.5%, respectively, before the initiation of the specific antibiotic or antimycotic treatment. Minor local adverse events were infrequently observed in both groups (1.1% and 2.0% of patients with Fitostimoline and benzydamine hydrochloride, resp.). No serious adverse events were observed during the study. A limitation of the study can be the absence of a placebo group; moreover this kind of study design has been judged as unethical. Additionally, the bacteriological test has not been repeated in the BV patients at the end of treatments, being the regression of clinical symptoms itself suggestive for the normalization of the vaginal flora.

In conclusion, Fitostimoline (vaginal cream, ovules, vaginal washing) was demonstrated to be an effective and well-tolerated treatment suitable for the topical management of bacterial vaginosis. Since BV is still characterized by a high recurrence rate [10], the long-term therapeutic effect of topical *Triticum vulgare* extract (Fitostimoline) should be the subject of future clinical evaluation.

## Figures and Tables

**Table 1:** Demographic and baseline clinical characteristics (ITT population). If not otherwise stated, entries are mean and 95% CI.

	Fitostimoline Cream + vaginal washing (*n* = 95)	Fitostimoline Ovules + vaginal washing (*n* = 97)	Pooled Fitostimoline (*n* = 192)	Tantum Rosa Cream + vaginal washing (*n* = 99)
Demographics				

Age [year]	36.9 [34.5–39.4]	36.3 [33.7–38.9]	36.6 [34.8–38.4]	36.4 [34.0–38.7]
Weight [kg]	63.6 [61.4–65.9]	64.0 [61.7–66.3]	63.8 [62.2–65.4]	63.2 [61.2–65.3]
Height [cm]	163 [162–164]	162 [161–163]	163 [162–163]	163 [162–164]
BMI [kg/m^2^]	23.8 [23.0–24.7]	24.2 [23.5–25.1]	24.0 [23.4–24.6]	23.8 [23.0–24.5]
Caucasian race *n* [%]	92 (96.9%)	95 (97.9%)	187 (97.4%)	98 (99.0%)

Obstetrics and gynaecologic				

Days from the last menstrual cycle	10.1 [9.4–10.7]	9.4 [8.7–10.0]	9.7 [9.2–10.2]	9.5 [8.8–10.2]
Use of oral contraceptives *n* [%]	13 (13.7%)	13 (13.4%)	26 (13.5%)	14 (14.1%)
Years from oral contraceptive start	0.5 [0.0–1.0]	0.5 [0.0–1.0]	0.5 [0.1–0.9]	0.5 [0.2–0.7]
Previous pregnancies *n* [%]	55 (57.9%)	52 (53.6%)	107 (55.7%)	63 (63.6%)
Number of previous pregnancies	1.4 [1.1–1.7]	1.5 [1.1–1.9]	1.5 [1.2–1.7]	1.6 [1.3–2.0]

Risk factors for bacterial vaginosis				

Regular sexual activity *n* [%]	87 (91.6%)	89 (91.8%)	176 (91.7%)	92 (92.9%)
Exposure to chemicals* and vaginal douching *n* [%]	13 (13.7%)	14 (14.4%)	27 (14.1%)	11 (11.1%)
Use of tight or synthetic clothes *n* [%]	30 (31.6%)	33 (34.0%)	63 (32.8%)	32 (32.3%)
Anxiety and depression *n* [%]	16 (16.8%)	14 (14.4%)	30 (15.6%)	14 (14.1%)

pH and Whiff-test				

pH assessment *n* [%]	31 (32.6%)	29 (29.9%)	60 (31.3%)	32 (32.3%)
pH value	5.6 [5.4–5.8]	5.5 [5.3–5.7]	5.5 [5.4–5.7]	5.5 [5.3–5.7]
Whiff-test performed *n* [%]	30 (31.6%)	32 (33.0%)	62 (32.3%)	31 (31.3%)

Vaginal swab				

Swab performed *n* [%]	95 (100.0%)	97 (100.0%)	192 (100.0%)	99 (100.0%)
Specific pathogens growth *n* [%]	51 (53.7%)	52 (53.6%)	103 (53.6%)	54 (54.5%)
Specific treatment *n* [%]	33 (34.7%)	28 (28.9%)	61 (31.8%)	30 (30.3%)

*Scented soaps, feminine hygiene sprays, and cleansers.

**Table 2:** Primary efficacy variable. Number and percentage of patients with therapeutic success.

Therapeutic success	PP population		ITTr population	
	Pooled Fitostimoline	Tantum Rosa	Pooled Fitostimoline	Tantum Rosa
Number (%) of patients	82 (98.8%)	39 (92.9%)	135 (82.8%)	66 (78.6%)
Difference (95% CI) Fitostimoline-Tantum Rosa	5.94 [−2.20, 14.07]		4.25 [−6.26, 14.76]	

**Table 3:** Outcome of signs and symptoms of vulvovaginitis and total symptom score (TSS) with Fitostimoline (pooled group) and Tantum Rosa. Entries are expressed as number of patients and percentage.

	PP population		ITTr population	
	Pooled Fitostimoline	Tantum Rosa	Pooled Fitostimoline	Tantum Rosa
Resolution	82 (98.8%)	39 (92.8%)	135 (82.8%)	66 (78.6%)
Improvement	0 (0%)	1 (2.4%)	8 (4.9%)	5 (6.0%)
Failure	1 (1.2%)	2 (4.8%)	20 (12.3%)	13 (15.5%)
